# Extrapelvic Urinary Extravasation Caused by Retroperitoneal Metastasis of Pulmonary Squamous Cell Carcinoma: A Case Report

**DOI:** 10.7759/cureus.102774

**Published:** 2026-02-01

**Authors:** Manato Taguchi, Takumi Kiwamoto, Shun Kagaya, Toshihiro Shiozawa, Nobuyuki Hizawa

**Affiliations:** 1 Department of Pulmonary Medicine, University of Tsukuba, Tsukuba, JPN; 2 Department of Pulmonary Medicine, University of Tsukuba, Ibaraki, JPN; 3 Department of Diagnostic and Interventional Radiology, University of Tsukuba Hospital, Tsukuba, JPN

**Keywords:** lung cancer, malignant ureteral obstruction, perirenal urine leakage, retroperitoneal metastasis, spontaneous urinary extravasation, squamous cell carcinoma, urinary extravasation

## Abstract

A 52-year-old man with pulmonary squamous cell carcinoma, clinically staged as cTxN3M1c (stage IVB), received four cycles of first-line chemotherapy with cisplatin plus TS-1 followed by seven cycles of second-line nivolumab. Subsequent imaging revealed progressive enlargement of cervical lymph nodes, left adrenal metastasis, and retroperitoneal metastases. Over several days, he developed left flank and back pain and presented to the emergency department.

An emergent contrast-enhanced abdominal computed tomography (CT) demonstrated contrast extravasation from the upper left ureter, left ureteral obstruction caused by retroperitoneal metastasis, and mild hydronephrosis. He was diagnosed with extrapelvic urinary extravasation and underwent placement of a polymeric double-J ureteral stent. His pain resolved promptly thereafter, allowing initiation of third-line chemotherapy.

Extrapelvic urinary extravasation due to retroperitoneal dissemination of pulmonary squamous cell carcinoma is extremely rare, and no prior cases were identified in our literature search. Nevertheless, recognizing this condition as a potential differential diagnosis for flank or back pain during disease relapse may facilitate early intervention, support continuation of systemic therapy, and ultimately improve quality of life.

## Introduction

Urinary extravasation is an uncommon condition characterized by the radiological demonstration of urine or contrast material outside the renal collecting system. In most cases, it is associated with trauma, iatrogenic manipulation, or obstructive pathology [1,2]. When extravasation occurs in the absence of recent instrumentation, trauma, prior surgery, destructive renal disease, or external compression, it is referred to as spontaneous urinary extravasation [1-3]. Spontaneous urinary extravasation is thought to occur when impaired urinary flow elevates intrapelvic pressure, producing microscopic tears in the urothelium at the fornix, through which urine seeps into the peripelvic or perirenal fat. The fornix is the most common site of rupture, followed by the upper ureter when pressure exceeds a critical level reported from 20 to 75 mmHg [4]. Urinary leakage may subsequently lead to the formation of a urinoma [5]. On imaging, the delayed phase of contrast-enhanced computed tomography (CT) scan is particularly valuable, as the distribution of contrast leakage often allows localization of the rupture site [1]. Most reported cases involve spontaneous rupture associated with ureteral calculi; in contrast, cases caused by malignant ureteral obstruction are relatively uncommon [6].

Here, we describe an extremely rare case of urinary extravasation caused by ureteral obstruction from retroperitoneal dissemination of pulmonary squamous cell carcinoma during systemic chemotherapy. Ureteral obstruction caused by lung cancer metastasis is itself uncommon [7], and to our knowledge, no previous reports have described urinary extravasation secondary to retroperitoneal metastasis of pulmonary squamous cell carcinoma. With the increasing use of immunotherapy and molecular targeted agents, survival among patients with lung cancer has improved, and clinicians may encounter this condition more frequently. It should therefore be considered in the differential diagnosis of abdominal or flank pain in patients with advanced lung cancer.

## Case presentation

**Figure 1:**
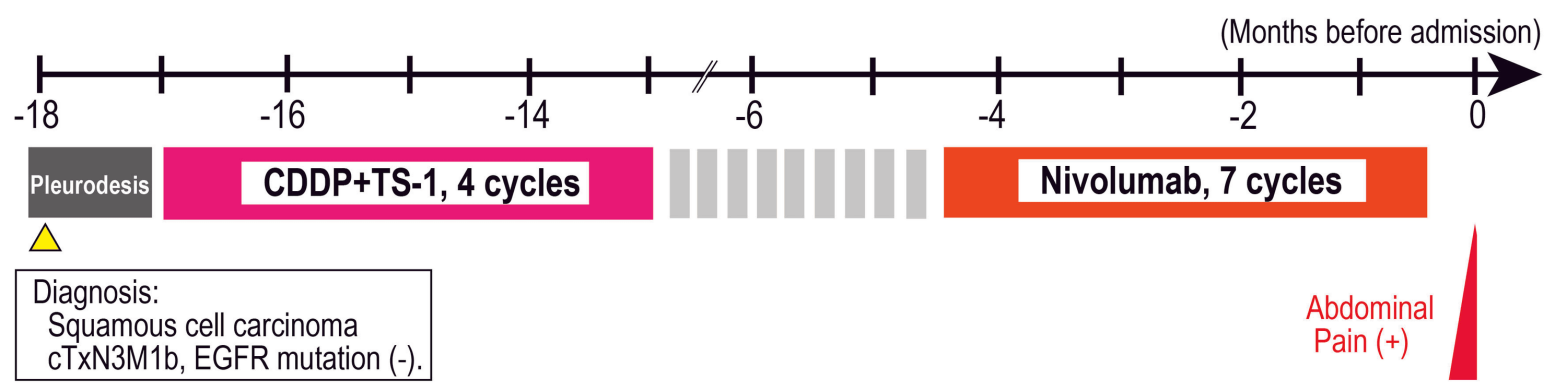
Clinical course Clinical course diagram showing the timeline from the initial presentation 18 months prior to admission through diagnosis and the administration of chemotherapy up to second-line treatment, culminating in the onset of abdominal pain. EGFR - epidermal growth factor receptor

A 52-year-old man presented to the emergency department with progressively worsening low back pain. He had been undergoing treatment for pulmonary squamous cell carcinoma for 18 months (Figure 1).

At age 50, he had been diagnosed with pulmonary squamous cell carcinoma, clinically staged as cTxN3M1c stage IVB (metastases to the right iliac bone, left perigastric lymph nodes, and malignant pleural effusion), with no epidermal growth factor receptor (EGFR) gene mutation detected by PCR-based assay. He received four cycles of first-line chemotherapy with cisplatin plus TS-1, achieving stable disease as the best response. However, the disease recurred eight months after completion of treatment, and seven cycles of nivolumab were administered as second-line therapy. The best response to second-line treatment was also stable disease. An evaluation CT performed one week prior to presentation revealed enlargement of para-aortic lymph nodes in the suprarenal region, left adrenal metastasis, newly identified retroperitoneal metastases, and vertebral bone metastases; therefore, initiation of third-line therapy was being planned. At the time of imaging, no evident hydronephrosis was observed. In addition, worsening cancer-related pain associated with malignant pleuritis had required initiation of regular oral oxycodone one month prior, in addition to etodolac.

Several days before admission, his left low back pain worsened despite increasing the regular dose of oxycodone and using additional rescue doses, eventually resulting in pain severe enough to impair his ability to stand. This prompted an emergency department visit. At presentation, he was alert, with a low-grade fever (37.4°C), while his blood pressure, heart rate, and oxygenation were stable. The Eastern Cooperative Oncology Group (ECOG) performance status was 1. His abdomen was flat and soft, but he had severe spontaneous pain in the left flank, making even light palpation intolerable. Laboratory evaluation showed mild inflammatory changes (white blood cells 8400/μL with 82.5% segmented neutrophils, C-reactive protein 2.26 mg/dL), while renal function remained preserved (blood urea nitrogen 10.2 mg/dL, creatinine 0.86 mg/dL, Table 1).

**Table 1:** Laboratory findings on admission Comprehensive laboratory data obtained at the time of hospital admission demonstrated a slight elevation of neutrophil and CRP. Reference ranges are shown in the rightmost column for comparison. WBC - white blood cell; Neu - neutrophil; Lym - lymphocyte; Eos - eosinophil; Baso - basophil; RBC - red blood cell; Hb - hemoglobin; Hct - hematocrit; Plt - platelet; Alb - albumin; AST - aspartate aminotransferase; ALT - alanine aminotransferase; LDH - lactate dehydrogenase; ALP - alkaline phosphatase; γ-GTP - gamma‑glutamyl transpeptidase; T-Bil - total bilirubin; Na - sodium; Cl - chloride; K - potassium; BUN - blood urea nitrogen; Cre - creatinine; eGFR - estimated glomerular filtration rate; CRP - C-reactive protein; Ca - calcium

Parameter (Unit)	Value	Reference range
Blood test		
WBC (/μL)	8400	4500-9000
Neu (%)	89.9	49-62
Lym (%)	9.7	25-45
Eos (%)	0.2	1-5
Baso (%)	0	0-1
RBC (×10⁴/µL)	430	427-570
Hb (g/dL)	13.3	14-18
Hct (%)	38.7	40-52
Plt (×10⁴/µL)	25.1	15-35
Alb (g/dL)	3.5	3.8-5.3
AST (U/L)	30	8-38
ALT (U/L)	13	4-44
LDH (U/L)	206	106-211
ALP (U/L)	273	104-338
γ-GTP (IU/L)	26	12-63
T-Bil (mg/dL)	0.6	0.3-1.2
Na (mEq/L)	138	135-147
Cl (mEq/L)	102	98-108
K (mEq/L)	4.7	3.6-5
BUN (mg/dL)	10.2	8-20
Cre (mg/dL)	0.86	0.61-1.04
eGFR (mL/min)	73.6	60-100
CRP (mg/dL)	2.26	0-0.2
Ca (mg/dL)	8.9	8.6-10.1

Chest radiography showed right-dominant pleural effusion due to malignant pleuritis, without significant interval worsening compared to prior CT, and no free air was detected. An upright abdominal radiograph revealed no abnormalities. Based on these findings, inadequate pain control due to progression of the primary disease was suspected, and he was admitted for analgesia. 

On hospital day two, however, he developed a fever of 39.3°C and worsening left back pain. Although chills or rigors were absent, left costovertebral angle tenderness and rebound tenderness were present. Laboratory testing showed further inflammatory elevation (white blood cells 10,200/μL with 80.2% segmented neutrophils, C-reactive protein 7.88 mg/dL, Table 2), although urinalysis was normal. Blood and urine cultures obtained at that time were negative, and urine cytology revealed no malignant cells.

**Table 2:** Laboratory findings on hospital day two Comprehensive laboratory data obtained at the time of hospital day two, demonstrating further elevation of WBC and CRP. Reference ranges are shown in the rightmost column for comparison. WBC - white blood cell; Neu - neutrophil; Lym - lymphocyte; Eos - eosinophil; Baso - basophil; RBC - red blood cell; Hb - hemoglobin; Hct - hematocrit; Plt - platelet; Alb - albumin; AST - aspartate aminotransferase; ALT - alanine aminotransferase; LDH - lactate dehydrogenase; ALP - alkaline phosphatase; γ-GTP - gamma‑glutamyl transpeptidase; T-Bil - total bilirubin; Na - sodium; Cl - chloride; K - potassium; BUN - blood urea nitrogen; Cre - creatinine; eGFR - estimated glomerular filtration rate; CRP - C-reactive protein; Ca - calcium

Parameter (Unit)	Value	Reference Range
Blood test		
WBC (/μL)	10200	4500-9000
Neu (%)	90.1	49-62
Lym (%)	9.4	25-45
Eos (%)	0.4	1-5
Baso (%)	0.1	0-1
RBC (×10⁴/µL)	421	427-570
Hb (g/dL)	12.8	14-18
Hct (%)	38	40-52
Plt (×10⁴/µL)	25.6	15-35
Alb (g/dL)	3.1	3.8-5.3
AST (U/L)	40	8-38
ALT (U/L)	12	4-44
LDH (U/L)	322	106-211
ALP (U/L)	253	104-338
γ-GTP (IU/L)	24	12-63
T-Bil (mg/dL)	0.9	0.3-1.2
Na (mEq/L)	136	135-147
Cl (mEq/L)	100	98-108
K (mEq/L)	4,3	3.6-5
BUN (mg/dL)	10	8-20
Cre (mg/dL)	0.96	0.61-1.04
eGFR (mL/min)	65.3	60-100
CRP (mg/dL)	7.88	0-0.2
Ca (mg/dL)	8.5	8.6-10.1
Urinalysis		
pH	6.5	4.6-7.5
Specific gravity	1.009	1.015-1.025
Protein	negative	negative
Glucose	negative	negative
Occult blood	negative	negative
Nitrite	negative	negative
Urine WBC (qualitative)	negative	negative

**Figure 2:**
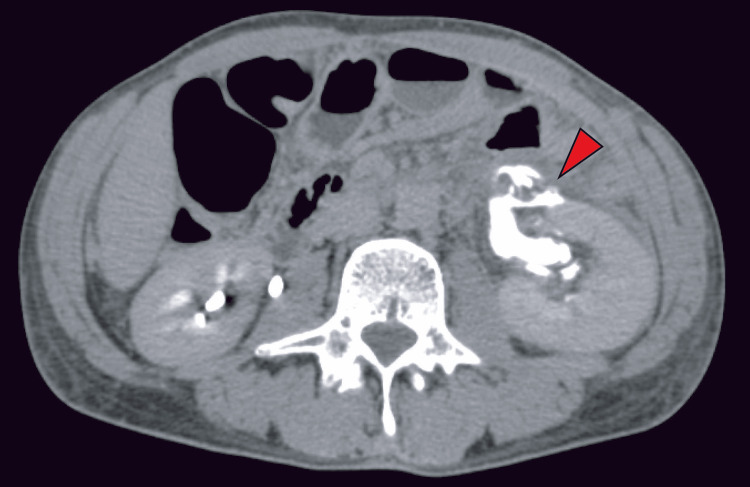
Delayed-phase contrast-enhanced abdominal CT on hospital day two Red arrows indicate the site of contrast medium extravasation. Delayed-phase contrast-enhanced CT demonstrated extravasation of contrast material from the left renal pelvis into the left perirenal space.

**Figure 3:**
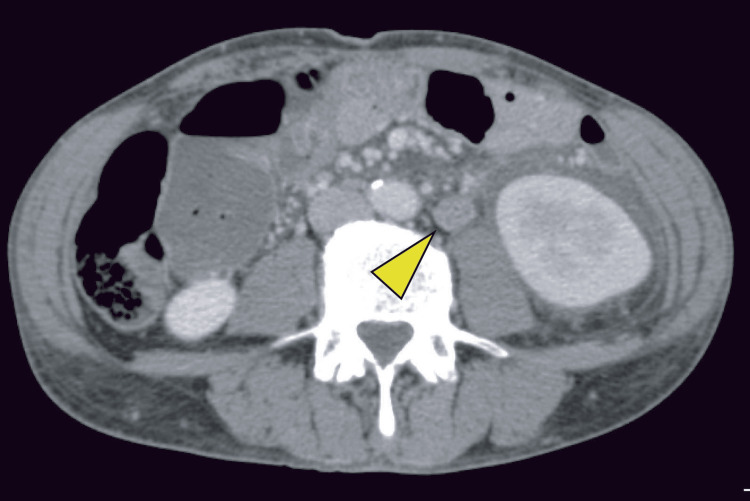
Contrast-enhanced abdominal CT on hospital day two Yellow arrows indicate newly developed retroperitoneal metastasis adjacent to the left ureter.

Contrast-enhanced CT demonstrated urinary extravasation from the left upper ureter in the delayed phase (Figure 2). In addition, a soft-tissue density suggestive of tumor progression was observed adjacent to the left ureter (Figure 3).

**Figure 4:**
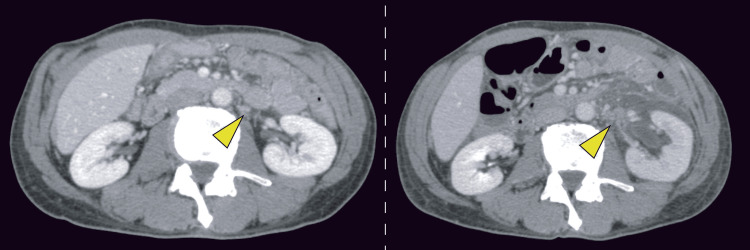
Contrast-enhanced abdominal CT CT obtained one week before admission (left panel) and on hospital day two (right panel). Yellow arrows indicate retroperitoneal metastatic lesions.

A CT scan obtained one week prior to admission had revealed left adrenal metastasis and retroperitoneal metastatic lesions (Figure 4); however, these lesions had progressed, and hydronephrosis had subsequently developed.

Based on these findings, the patient was diagnosed with urinary extravasation secondary to ureteral obstruction caused by encasement of the ureter by retroperitoneal tumor invasion resulting from progression of lung squamous cell carcinoma.

**Figure 5:**
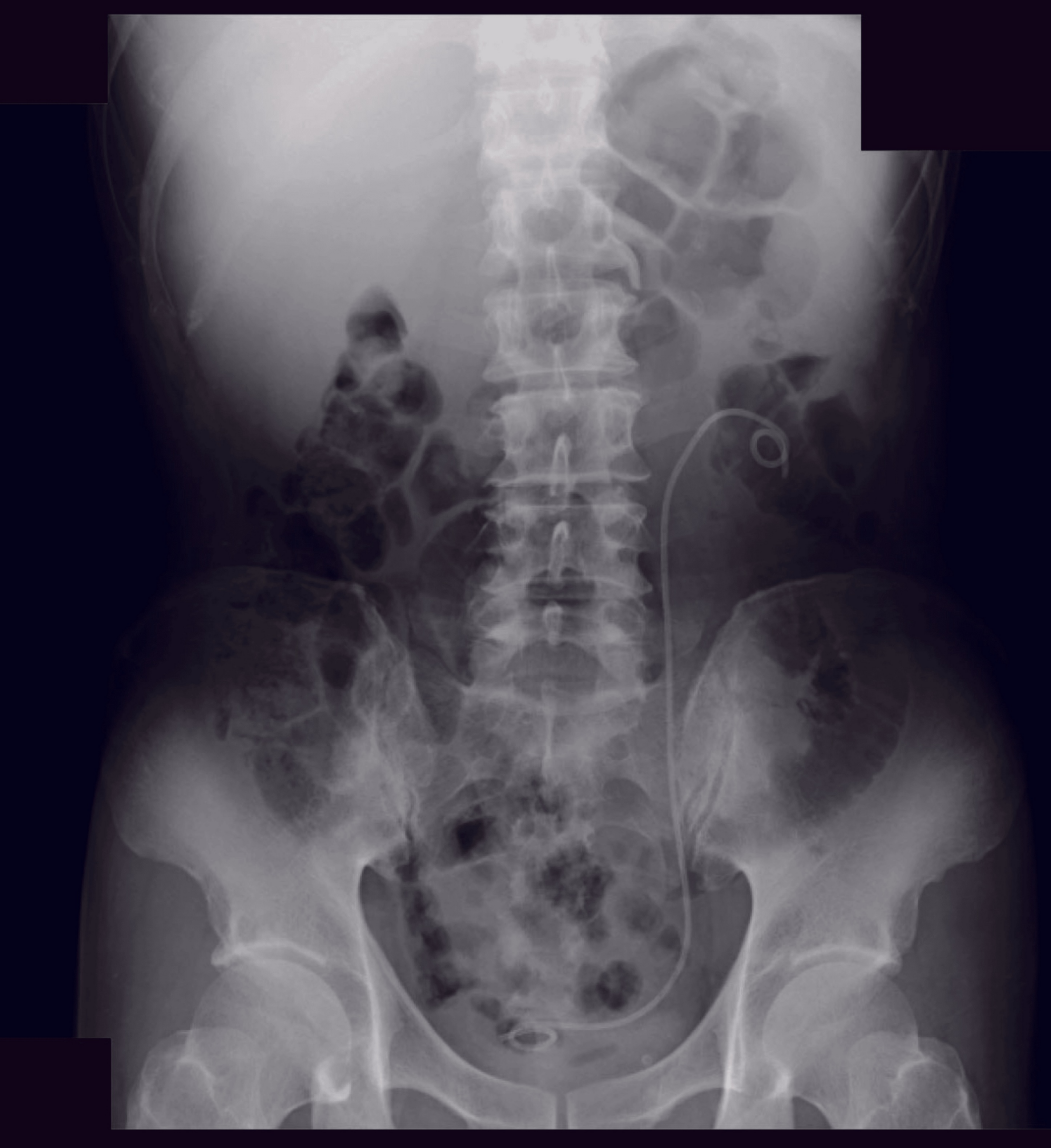
Plain abdominal radiograph obtained after placement of a double-J ureteral stent

Following diagnosis, the patient was treated with antibiotics and underwent placement of a polymeric double-J ureteral stent (Figure 5), resulting in substantial symptom improvement. Combination chemotherapy with cisplatin and vinorelbine as third-line therapy was initiated on hospital day 19.

**Figure 6:**
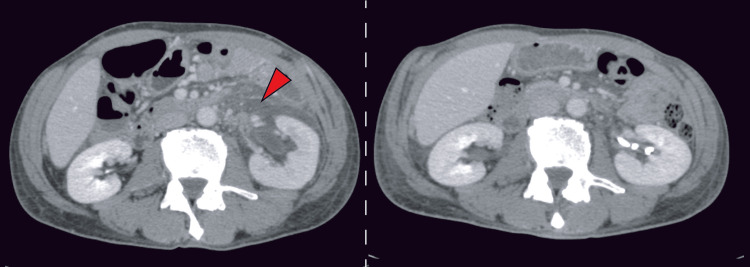
Contrast-enhanced abdominal CT after treatment Contrast-enhanced abdominal CT on hospital day two (left panel) and one month after ureteral stent placement (right panel). The perirenal fluid collection around the left kidney (red arrow) and hydronephrosis observed on hospital day two showed marked improvement after stent placement.

A follow-up CT scan performed one month after stent placement showed improvement in both the perirenal fluid collection and hydronephrosis, (Figure 6), allowing discharge. After discharge, the ureteral stent was left in place, and third-line chemotherapy was continued. Thereafter, no deterioration in urinary outflow or renal function was observed, and stent exchanges performed every three months were uneventful; therefore, a polymeric double-J ureteral stent was continuously used. Following completion of third-line therapy, the patient was managed with observation alone for three months, after which disease recurrence was noted. Fourth-line therapy with docetaxel plus ramucirumab, fifth-line therapy with irinotecan monotherapy, and sixth-line therapy with amrubicin monotherapy were administered but were ineffective. He died of disease progression one year after discharge.

## Discussion

In the present case, retroperitoneal dissemination of lung squamous cell carcinoma was considered to have caused ureteral compression, leading to ureteral obstruction. In a report by Gershman et al. in 2011, urolithiasis was identified as the most frequent cause of renal forniceal rupture, accounting for approximately 74.1% of cases, whereas malignant extrinsic ureteric compression was relatively uncommon, comprising only 8.3% [6]. Furthermore, Artiles et al. reported that the most frequent causes of malignant ureteral obstruction were prostate (17.6%), bladder (16.5%), and rectal cancer (11.7%), while lung cancer was rare, accounting for only 1.6% of cases [7]. Only a single case of retroperitoneal metastasis from pulmonary squamous cell carcinoma has been reported [8]. To the best of our knowledge, no cases of urinary extravasation caused by lung squamous cell carcinoma have been reported. Araya et al. recently reported three cases of malignant ureteral obstruction caused by retroperitoneal metastases from lung adenocarcinoma [9]. In their series, two patients harbored EGFR mutations, suggesting that prolonged survival achieved with targeted therapies may allow lung cancer to metastasize to uncommon sites. Although the present case was squamous cell carcinoma, disease control was maintained for a relatively long period for stage IV squamous cell carcinoma, indicating that similar conditions predisposing to unusual metastatic patterns may have been present. 

Imaging studies are useful for the diagnosis of urinary extravasation. Contrast-enhanced CT can demonstrate perirenal fluid collection and ureteral dilatation. In the delayed phase of contrast-enhanced CT, the site of rupture can often be identified based on the extent of contrast leakage; however, when urinary leakage is accompanied by an inflammatory response, patients may present with fever and pain, making differentiation from acute pyelonephritis or renal colic difficult and potentially delaying diagnosis [1]. In the present case, CT revealed perirenal fluid collection around the left kidney, leading to the diagnosis of urinary extravasation. 

The management of urinary extravasation primarily consists of treatment of the underlying cause and urinary tract drainage [10]. Because urinary tract obstruction can lead to irreversible renal damage and may preclude the administration of systemic therapy; therefore, prompt decompression by ureteral stent placement or percutaneous nephrostomy is essential to preserve renal function and enable continuation of cancer treatment [11-13]. In the present case, worsening retroperitoneal metastases from lung cancer during chemotherapy led to the development of urinary extravasation, and urinary drainage was therefore achieved by placement of a ureteral polymeric double-J ureteral stent. As a result, the perirenal fluid collection decreased, symptoms improved, and the patient was able to proceed to third-line therapy. The prognosis of lung squamous cell carcinoma has improved with the widespread use of immunotherapy and the expansion of treatment options [14]. This improvement in survival should be considered a potential factor increasing the incidence of rare complications at distant metastatic sites, such as those with limited responsiveness to chemotherapy, as observed in the present case. 

## Conclusions

We reported a case of extrapelvic urinary extravasation caused by retroperitoneal metastasis of pulmonary squamous cell carcinoma. To our knowledge, no previous cases of extrapelvic urinary extravasation associated with pulmonary squamous cell carcinoma have been reported. With improvements in the long-term prognosis of pulmonary squamous cell carcinoma, clinicians should consider extrapelvic urinary extravasation secondary to retroperitoneal metastasis as a potential cause when patients with an expected long-term survival present with acute abdominal symptoms.
